# Tree species admixture increases ecosystem service provision in simulated spruce- and beech-dominated stands

**DOI:** 10.1007/s10342-022-01474-4

**Published:** 2022-07-02

**Authors:** Reinhard Mey, Jürgen Zell, Esther Thürig, Golo Stadelmann, Harald Bugmann, Christian Temperli

**Affiliations:** 1 Forest Resources and Management, Swiss Federal Institute for Forest, Snow and Landscape Research WSL, 8903 Birmensdorf, Switzerland; 2 Forest Ecology, Institute of Terrestrial Ecosystems, Department of Environmental Systems Science, ETH Zurich, 8092 Zurich, Switzerland

**Keywords:** Adaptive forest management, Climate change, Ecosystem services, Empirical stand simulator, Sensitivity analysis, Trade-offs and synergies

## Abstract

Climate-adaptive forest management aims to sustain the provision of multiple forest ecosystem services and biodiversity (ESB). However, it remains largely unknown how changes in adaptive silvicultural interventions affect trade-offs and synergies among ESB in the long term. We used a simulation-based sensitivity analysis to evaluate popular adaptive forest management interventions in representative Swiss low- to mid-elevation beech- and spruce-dominated forest stands. We predicted stand development across the twenty-first century using a novel empirical and temperature-sensitive single-tree forest stand simulator in a fully crossed experimental design to analyse the effects of (1) planting mixtures of Douglas-fir, oak and silver fir, (2) thinning intensity, and (3) harvesting intensity on timber production, carbon storage and biodiversity under three climate scenarios. Simulation results were evaluated in terms of multiple ESB provision, trade-offs and synergies, and individual effects of the adaptive interventions. Timber production increased on average by 45% in scenarios that included tree planting. Tree planting led to pronounced synergies among all ESBs towards the end of the twenty-first century. Increasing the thinning and harvesting intensity affected ESB provision negatively. Our simulations indicated a temperature-driven increase in growth in beech- (+ 12.5%) and spruce-dominated stands (+ 3.7%), but could not account for drought effects on forest dynamics. Our study demonstrates the advantages of multi-scenario sensitivity analysis that enables quantifying effect sizes and directions of management impacts. We showed that admixing new tree species is promising to enhance future ESB provision and synergies among them. These results support strategic decision making in forestry.

**Supplementary Information:**

The online version contains supplementary material available at 10.1007/s10342-022-01474-4.

## Introduction

Adaptive forest management is pivotal to sustain the provision of multiple ecosystem services and biodiversity (ESB) as well as forest multi-functionality in the face of climate change (Bolte et al. 2009; Keenan 2015; Millar et al. 2007; Spathelf et al. 2018). Adaptive management strategies include changing the tree species composition and promoting species and stand structural diversity to increase forest resilience to disturbances (Bolte et al. 2009; Messier et al. 2019; Pretzsch et al. 2017). Devising adaptive and sustainable forest management requires in-depth knowledge on the effects of silvicultural interventions on individual and multiple ESB. In particular, further research on how adaptive strategies will change mutual relations (trade-offs and synergies) among ESB in the long term is required (e.g. Sousa-Silva et al. 2018).

Proactive adaptation is the application of specific silvicultural interventions that aim to ensure the long-term provision of forest ESB based on information on potential future developments such as projected climatic shifts and their impact on tree growth (Bolte et al. 2009; Spathelf et al. 2018; Yousefpour et al. 2017). Diverse silvicultural interventions have been investigated to achieve proactive adaptation. Planting of tree species that are adapted to future climatic conditions (Hof et al. 2017) and thinning of climatically unsuited species (D’Amato et al. 2013; Elkin et al. 2015) reduce climate change-related risks and foster the transition to a more resistant and resilient species composition. Promoting species diversity through planting and silvicultural interventions such as thinning of dominant tree species may spread the risk of climate change-induced stress across multiple species (Vitali et al. 2018) and increase functional diversity and the resilience against disturbances (D’Amato et al. 2013; Hof et al. 2017). It may promote the provision of multiple ESB (Schuler et al. 2017) and stand productivity (Pretzsch et al. 2010; Zhang et al. 2012). Shortening of the rotation period may mitigate the risk of windthrow (Meilby et al. 2001) and maintain high productivity (D’Amato et al. 2010).

Yet, adaptive forest management practices also bear risks for ESB provision (Hof et al. 2017) such as lower volume increment (Hanewinkel and Pretzsch 2000) and thus reduced harvest revenue. In many simulation studies, sometimes severe trade-offs between timber production and regulating services such as carbon storage, mitigation of natural hazards and sustaining biodiversity were identified (e.g. Lafond et al. 2017; Turner et al. 2013). Thus, an in-depth analysis based on rigorous tools is needed to assess ESB under varying adaptive management regimes and climate scenarios.

To assess the future impacts of proactive adaptation on the provision of ESB (i.e. trade-offs and synergies), dynamic forest development models are often used (Mina et al. 2017; Temperli et al. 2012) jointly with ESB indicator frameworks (Blattert et al. 2017). Ideally, such models are based on individual trees to reflect detailed management practices, and they should consider the simultaneous effects of competition, stand development, local site conditions, stand density, thinning, mixture and climate on all demographic processes.

Scenario analyses based on dynamic forest simulation models allow for quantifying the effects of changes in the environment (e.g. climate) and in management strategies on forest development over decades to centuries, and they are highly suitable to support decision making. For operational forest management, forest stands are the major target. For forest models to be accurate at the stand level, high data requirements, parameterisation and calibration efforts and computing time are required. Hence, to date most studies providing detailed stand-scale predictions are typically based on case studies comprising specific sites or relatively small forest landscape (e.g. Mina et al. 2017) and thus lack representativity at the regional to country scale. However, upscaling from the stand level is key to understand the broader consequences of management practices and to generalise scientific results (Reyer et al. 2015). Stand descriptions derived from National Forest Inventory (NFI) data based on novel methods (Mey et al. 2021) allow for initialising individual-tree stand simulators with regionally representative data, and thus for bridging the gap between stand-scale accuracy and regional-scale representativity.

Scenario analyses usually investigate future forest trajectories based on a single or a few management storylines that have specific goals, such as the promotion of biodiversity or maximum timber production. These storylines are often specified as a combination of silvicultural interventions including planting and various tending, thinning and harvesting prescriptions based on expert knowledge or literature reviews (Duveneck and Scheller 2015; Temperli et al. 2012). However, single-scenario approaches only examine a small set of the theoretically possible combinations of silvicultural interventions at specific intensities. A simulation-based sensitivity analysis that evaluates the effects of multiple silvicultural interventions and intervention intensities on ESB provision in a crossed experimental design is necessary to quantify effect sizes and directions of the individual silvicultural interventions, identify interactions between silvicultural interventions, and to reveal trade-offs and synergies between ESB over time.

The aim of the present study is to systematically evaluate the effects of adaptive forest management interventions on ESB under climate warming in regionally representative, low- to mid-elevation beech (*Fagus sylvatica* L.)–and spruce (*Picea abies* (L.) Karst.)-dominated stands in Switzerland. We present a simulation-based sensitivity analysis that is both accurate at the stand scale and representative at the regional scale, and allows to quantify the effect sizes and effect directions of climate-adaptive silvicultural interventions. Specifically, we addressed the following questions:

- How do adaptive silvicultural interventions affect ESB provision under climate warming in representative pole stage and mature spruce- and beech-dominated stands?
- How do these interventions affect trade-offs and synergies between ESB?

## Material and methods

### Forest growth model SwissStandSim

All simulations were performed with SwissStandSim, an empirical, climate-sensitive individual-tree model developed for stand-scale predictions in Switzerland (Zell 2018; Zell et al. 2020). It is based on data from 374 Swiss Growth-And-Yield stands with an average area of ca. 2500 m^2^ (Forrester et al. 2019). The data span the period from 1902 to 2013. The sub-models for single-tree growth (in diameter and height), mortality, regeneration and harvesting are based on equations that have a semi-mechanistic interpretation. For example, growth is co-determined by the competitive effect of larger trees in the stand, stand density, and site variables such as nitrogen deposition. The growth models were cross-validated showing good prediction performances (Zell 2018).

Due to the direct integration of temporally varying temperature and precipitation variables in the equations, the model is climate sensitive. A ‘moisture index’ based on the ratio of precipitation to temperature is used to quantify drought (i.e. the inverse of water availability). The Growth-And-Yield stands used for model parameterisation cover a broad range of mean annual temperatures (0.2–10.2 °C). Therefore, tree growth and regeneration responses to changes in mean temperature as implied by climate scenarios are likely well captured by SwissStandSim. However, the Growth-And-Yield stands have been little affected by drought events in the past and they were re-measured at best every 5 years, so that potential short-term drought effects could not be included in the model. We thus expect simulated growth, regeneration and, in particular, mortality responses to drought under climate change to be underestimated and associated with a high degree of uncertainty. Hence, we focus on the effects of climate warming and do not account for potential drought effects.

A new light- and temperature-dependent ingrowth routine has been implemented based on light availability in the stands (Schumacher 2004) linked to ingrowth data from the Swiss NFI (details in Supporting Information S1 and Fig. S1). All stand-related and environmental explanatory variables are based on measured data and were parameterised separately for the eleven most abundant tree species groups in Switzerland (Zell et al. 2020). Simulations can be performed by varying the intensity, timing and type of thinning (e.g. thinning from below or above, crop tree management and plenter forest management, cf. Thrippleton et al. 2021a), and they deliver individual-tree and stand-specific outputs every 5 years (e.g. diameter at breast height (DBH), basal area, species composition and harvestable volume). These outputs are used for the analysis of ESB provision under forest management scenarios.

### Stand initialisation

**Fig. 1:**
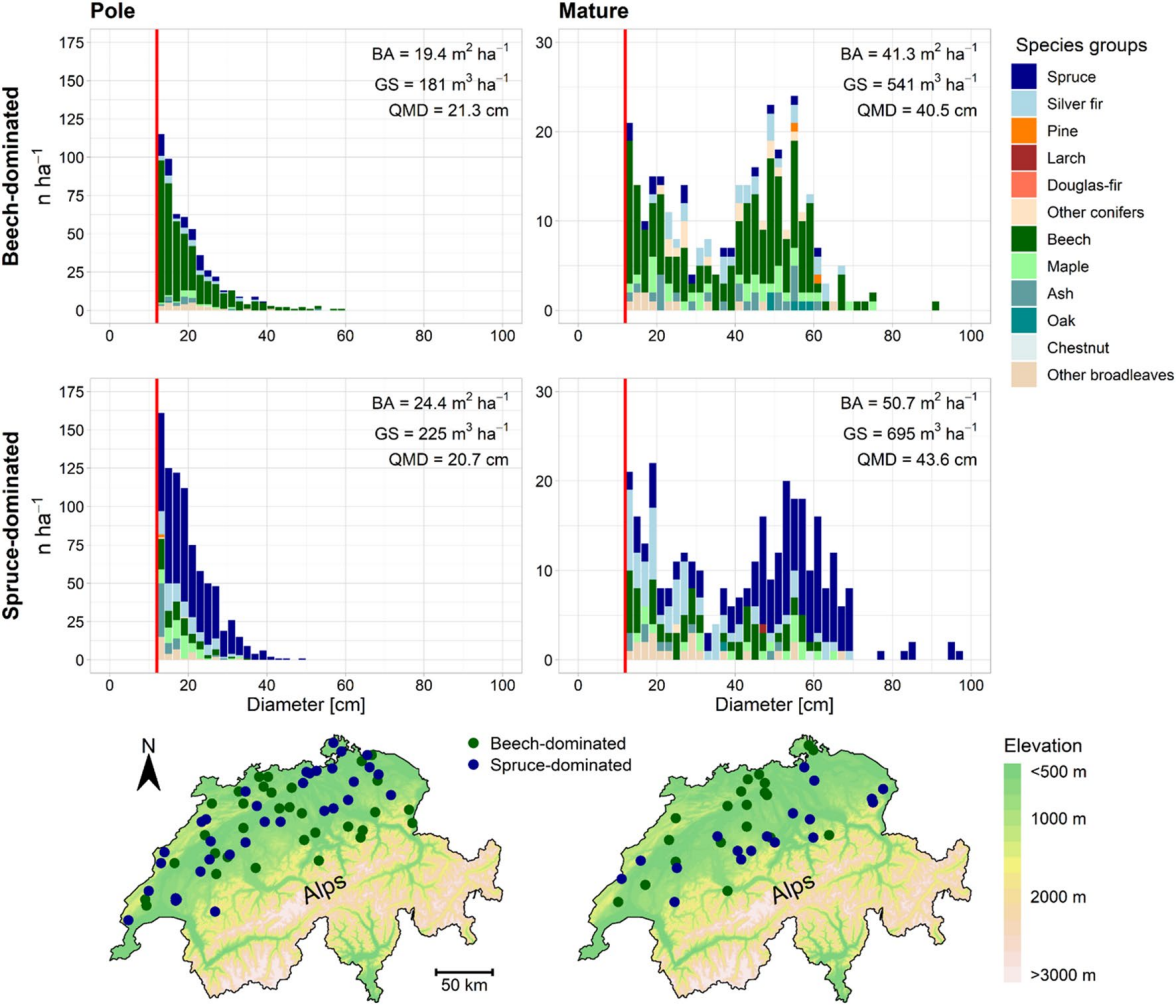
Example realisation of the initial stand descriptions (diameter distributions and species compositions) of beech- and spruce-dominated stands in the development stages pole and mature with the mean basal area (BA), mean growing stock (GS) and mean quadratic mean diameter (QMD) of the initial stands used in the simulation runs. Calliper threshold in red (at 12 cm). The maps of Switzerland show the location of the NFI4 plots used to derive the individual stand descriptions

To initialise the model with stand data, the approach by Mey et al. (2021) was used for deriving representative descriptions (i.e. diameter distribution and tree species composition, see Fig. 1) of beech- and spruce-dominated stands with an area of one hectare using data from the fourth Swiss NFI (NFI4; Brändli et al. 2020).

From the NFI sample plots north of the Alps and below 1200 m above sea level, we selected and classified 52 plots as beech-dominated (share of basal area of beech > 50%) and 52 plots as spruce-dominated forest (share of basal area of spruce > 80%). The plots were further classified as “pole stage” and “mature” based on the development stage determined by the field teams of the Swiss NFI. This selection of NFI plots was used to calculate median stand attributes (Table 1), which in turn were used to derive the diameter distributions and species compositions of generic stands that represent the sampled forest in the four classes.

**Table 1:** Overview of plot-based median statistics with the 5% and 95% quantile in brackets derived from NFI4 sample data and used to predict stand initialisations using the approach by Mey et al. (2021)

	Unit	Beech-dominated		Spruce-dominated	
Elevation	m a.s.l	655 (451, 1,026)		706 (435, 1,167)	
Mean winter temperature (1988–2017, Dec-Feb)	°C	1.13 (− 0.46, 1.65)		–0.72 (− 1.32, 1.66)	
Development stage	–	Pole	Mature	Pole	Mature
Modality of diameter distribution	–	Unimodal	Bimodal	Unimodal	Bimodal
Number of sample plots	–	32	20	34	18
Stems per hectare (> 12 cm)	n ha^−1^	545 (296, 945)	320 (149, 695)	835 (342, 1,585)	340 (138, 512)
Stems per hectare (> 0 cm)	n ha^−1^	2,827 (720, 10,088)	845 (384, 2,451)	1,464 (685, 7,582)	1,098 (355, 1,845)
Mean diameter	cm	7.21 (2.41, 22.5)	14.6 (6.38, 38.0)	15.1 (4.12, 25.7)	15.3 (9.13, 33.7)
Spread of (sampled) diameters	cm	31.0 (17.7, 73.0)	58.5 (43.7, 73.2)	27.0 (13.7, 52.4)	66.0 (48.4, 80.8)
Diameter 90%	cm	18.0 (4.10, 30.2)	51.0 (28.0, 67.7)	24.2 (13.5, 34.4)	50.5 (38.9, 65.3)
Diameter 10%	cm	1.50 (0.50, 15.6)	2.50 (0.50, 16.6)	5.50 (0.50, 17.6)	2.50 (0.50, 13.0)
Overstorey quadratic mean diameter	cm	–	49.9 (39.5, 60.6)	–	52.8 (42.4, 62.6)
Empirical rho	–	–	0.78 (0.38, 0.88)	–	0.76 (0.46, 0.89)

We distinguished samples featuring unimodal (one peak) and bimodal (two peaks) diameter distributions. The development stage was determined by the dominant diameter (mean diameter of the 100 trees with largest DBH, ddom) by the field teams in the Swiss NFI (pole = 12–30 cm ddom, mature > 50 cm ddom). The spread of the diameters is the maximum minus the minimum diameter of the trees of a sample. The overstorey quadratic mean diameter is the quadratic mean diameter of the trees in the overstorey of the bimodal samples determined by the approach of Mey et al. (2021). The empirical rho determines the share of the understorey in the bimodal samples. Further details are given in Mey et al. (2021)

While a unimodal Weibull function was used to represent the pole stage, a bimodal Weibull function was used for the mature stage (cf. Figure 1). However, in contrast to the approach from Mey et al. (2021), the species composition was predicted using a random forest approach (Breiman 2001), which showed better prediction performance (i.e. mean balanced accuracy (across all species) = 81.0%) than a multinomial logistic regression (mean balanced accuracy = 60.3%). The random forest is a decision tree-based nonlinear ensemble method that requires no assumptions about distributions, no data transformations, is robust against outliers, can handle imbalanced data and automatically performs a cross-validation (out-of-bag). However, in comparison with the multinomial logistic regression, the interpretability of the effects of the explanatory variables is reduced (cf. Mey et al. 2021).

In summary, we derived four stand descriptions for initialisation that are spatially representative for the Swiss region north of the Alps below 1200 m above sea level. These initial stand descriptions account for high natural regeneration of native silver fir, beech and other broadleaves in the study region, while secondary spruce forests are about to disappear (Brändli and Brang 2015; Brändli and Imesch 2015). For simulations, we only used trees with DBH > 12 cm since the light- and temperature-dependent ingrowth module was parameterised for this ingrowth threshold. These stands represent the initial state of the species composition and structure of the stands, which were then converted in the scenarios by different silvicultural interventions so that they were not necessarily dominated by spruce or beech after the first time step of the simulation.

### Scenario analysis

**Fig. 2:**
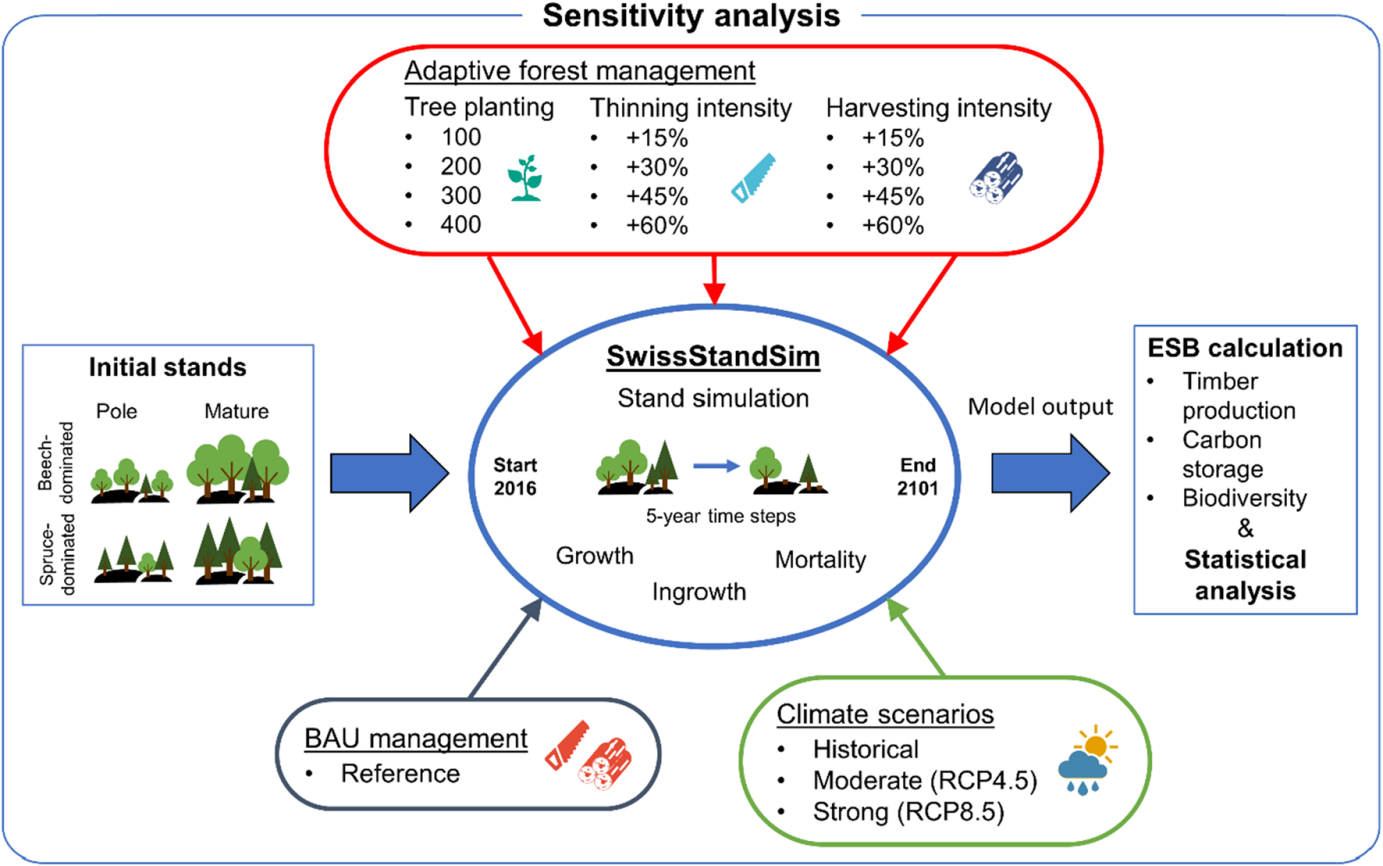
Simulation setup in the framework of a sensitivity analysis including three climate scenarios, the business as usual (BAU) management scenario (reference) and three adaptive silvicultural interventions with four levels per intervention, respectively (number of planted trees per hectare; percentual increase of thinning [across all diameter classes] and harvesting [target diameters] intensity of beech and spruce)

We used an empirically based representation of current management practices in Switzerland (BAU management) and reduced BAU management intensity by 25% as reference scenarios, for the reasons described below (Sect. Business as usual (BAU) management scenario). This reference scenario was modified with respect to tree planting and thinning (across all diameter classes) and harvesting intensities (target diameters, see Sect. Adaptive forest management below) to generate scenarios of adaptive silvicultural interventions. To quantify their effects and the effects of climate warming on ESB, we performed a sensitivity analysis with the gradually varied adaptive silvicultural interventions and three climate scenarios for each of the four initial stand descriptions (Fig. 2). Following a fully crossed experimental design, we simulated each combination of generic stand, reference and adaptive forest management as well as climate scenario (4 stands × 125 management variants × 3 climate scenarios = a total of 1500 scenarios) and replicated each scenario 20 times to account for the stochasticity in the growth, mortality and management sub-models.

An indicator framework was used to calculate ESB provision for each scenario, and the effects of management and climate on the provision of ecosystem services were quantified using ANOVA. Spearman rank correlation coefficients were used to quantify trade-offs and synergies among ESB. All analyses and visualisations were conducted with *R* version 4.0.3 (R Core Team 2020).

#### Business as usual (BAU) management scenario

A survey-weighted binomial GLM (Lumley 2010) was used to calculate the removal probabilities of single trees using the data of four consecutive inventories of the Swiss NFI (NFI1 to NFI4). The model (cf. S2) incorporates 12 explanatory variables that may be interpreted as proxies for timber revenue (tree species, diameter), accessibility of the plots (distance to forest road, slope, elevation), growth conditions (increment, basal area, number of stems), socio-economy (ecoregion, forest ownership) and disturbance events (storm). Additionally, the time between two consecutive inventories was used as an explanatory variable, which describes the effect of the harvest interval (i.e. 5, 10 or 15 years).

Over the four NFI inventory periods (1983–2017), the observed 10-year single-tree removal probability north of the Alps and below 1200 m is 21.8%. This corresponds well to other European forest inventory data summarised in Schelhaas et al. (2018), yielding a removal probability of 2.4% per year.^1^ Simulation runs, however, showed that a continuous 10-year removal probability of around 20% reduced the growing stock in the study area to around 250 m^3^ ha^−1^ and basal area to 20 m^2^ ha^−1^ in the long term (> 100 years). In contrast, today’s mean growing stocks north of the Alps are between 350 and 400 m^3^ ha^−1^. This overestimate of harvesting may be due to an overrepresentation of salvage cuttings following disturbance events in the NFI data, and perhaps also a general overexploitation of growing stocks on the Swiss Plateau (Stadelmann et al. 2016). To achieve growing stocks of around 350 m^3^ ha^−1^ in long-term simulations, we thus defined a BAU scenario (subsequently called “reference scenario”) by reducing the removal probability by 25% (Fig. S2). This reference was used for all comparisons to the scenarios of adaptive management.

The long-term application of the management model in a 10-year interval resulted in the uni- and bimodal diameter distributions of the generic stands to converge towards an inverse J-shaped distribution resembling continuous-cover forests (Fig. S3). A harvest interval of 10 years was used in all simulations, as this was approximately the interval the NFI plots have been sampled.

#### Adaptive forest management

Three adaptive forest management interventions were defined: a) planting of Douglas-fir, oak and silver fir, b) increased thinning intensity of climatically unsuited tree species to favour planted trees and natural regeneration, and c) increased intensity of target diameter tree harvest. The selection of silvicultural interventions was inspired by stakeholder workshops within the SessFor project (NRP 73, http://www.nfp73.ch/en/projects/forestry/ecosystem-services-in-forests), in which stakeholders from federal and cantonal administration (FOEN, KOK), private associations (Wald Schweiz, Holzindustrie Schweiz), the School of Agricultural, Forest and Food Sciences (HAFL), forest enterprise (Wagenrain), and conservation (Birdlife, Pro Natura, WWF) participated. To account for different degrees of adaptation, four levels per silvicultural intervention were defined (Fig. 2). Each combination of the four levels of the individual interventions was simulated. Planting was applied in addition to the reference scenario, and the increases of thinning (across all diameter classes) and harvesting (target diameters) intensity of beech and spruce were implemented by modifying the reference management.

- Planting of new trees

The goal of tree planting as an adaptive silvicultural intervention is to actively extend the tree species mixture by species that are expected to better cope with projected future climate conditions and thus to enhance forest resistance, or at least resilience. Note that simulating planting interventions served to investigate the effect of this management intervention on ESB provision and not to investigate the drought tolerance of planted species. In both forest types, a mixture of Douglas-fir, oak and silver fir was planted, which are considered to be more drought-tolerant than spruce and beech (Sáenz-Romero et al. 2017; Spiecker et al. 2019; Vitasse et al. 2019). In the beech-dominated stands, the species mix was even, while in the spruce-dominated stands Douglas-fir and oak accounted for 40% and silver fir for 20% of the trees, because silver fir is usually more abundant in low- to mid-elevation spruce-dominated stands (cf. Figure 1). The trees were assumed to grow in after 15 years of simulation (i.e. in 2031) with a DBH of 12 cm (± 0.5 cm SD to account for individual growth rates). Note that growth of the planted trees in the first 15 years was estimated using yield tables for Switzerland (Badoux 1983) reflecting optimum conditions. We did not distinguish growth rates of the individual species to give all planted trees the same starting conditions in 2031. This implicitly assumes tending interventions to reduce competition among planted saplings.

The four levels of planting intensity (Fig. 2) were chosen to approximate recommended densities at a stand age of 80 years; they factored in mortality due to competition and tending (see Ebert 1999). Depending on the development stage of the initial stand, tree plantings were supported by preparatory thinning (mature: 100% of the trees with DBH > 30 cm in 2016; pole stage: 50% of the trees with DBH > 12 cm in 2016, 30% > 20 cm in 2026 and 100% > 30 cm in 2031).

(b) Increased thinning intensity of climatically unsuited species

The aim of thinning climatically unsuited tree species with a higher intensity is to foster conversion towards a more climate change-adapted species composition. Hence, in the case of the spruce-dominated forest spruce, and in the case of the beech-dominated forest beech were considered as climatically unsuited. To achieve this active adaptation, the removal probability of spruce and beech derived from the reference management model was increased by 15%, 30%, 45% and 60%, respectively, over the entire simulation period.

(c) Increased harvesting intensity of target diameter trees

The goal of an increased harvesting intensity of target diameter trees is to shorten the rotation length and thus to reduce stand vulnerability to disturbances such as windthrow or bark beetle outbreaks. To shorten the rotation length, the removal probability of broadleaved trees with DBH ≥ 60 cm and conifers with DBH ≥ 50 cm was increased by 15%, 30%, 45%, and 60%, respectively, compared to the reference scenario over the entire simulation period.

#### Climate scenarios

The data for the historical climate scenario were randomly resampled from the reference period 1986–2015 provided by MeteoSwiss at the spatial resolution of the Swiss NFI grid (1.4 by 1.4 km, Brändli et al. 2020). In addition to the historical climate, SwissStandSim simulations were driven with two climate change scenarios (RCP4.5 and RCP8.5) from Brunner et al. (2019). They are based on the CH2018 climate scenarios (CH2018 2018), which in turn are based on Representative Concentration Pathways (RCP). The scenarios were downscaled and bias-corrected using quantile mapping (Feigenwinter et al. 2018). To obtain elevation-dependent point estimates on the Swiss NFI grid, the approach by Brunner et al. (2019) was used to further downscale the climate data using detrended inverse distance weighting and bilinear interpolation. Climate data (annual mean temperature, precipitation sum, moisture index and mean winter temperature (Dec-Feb)) from all NFI plots classified as beech-dominated and spruce-dominated forest (cf. Section Stand initialisation) were averaged on a 5-year interval to derive the three climate scenarios for the 5-year time steps of SwissStandSim. The RCP4.5 scenario featured an 0.83 K increase of annual mean temperature and a decrease of the annual precipitation sum by 20 mm compared to the historical climate (i.e. observations from 1986–2015), and was termed “moderate” scenario. The RCP8.5 scenario was termed “strong” with + 4.95 K and -221 mm (Fig. S5).

### Ecosystem services and biodiversity (ESB) analysis

The SwissStandSim model output (18 5-year time steps from 2016–2101) was used to calculate ten ESB indicators to characterise (1) timber production, (2) carbon storage and (3) biodiversity (Table 2). The timber production indicators include growing stock, harvested timber volume and volume increment. Following Blattert and Lemm (2018), 10% of the harvested volume was assumed to remain in the stand as residue and was added to the deadwood pool of the stands.

**Table 2:** Indicators to characterise ecosystem services and biodiversity (ESB) and their weighting factors. Specific decay rates were considered for deadwood (Mackensen et al. 2003) and harvested wood products (Thrippleton et al. 2021a)

ESB	Indicator	Unit	Weight
Biodiversity	Tree species diversity, Shannon index	–	0.15
	Structural diversity, Post-hoc index	–	0.15
	Deadwood volume	m^3^ ha^−1^	0.35
	Large living trees (habitat trees)	n ha^−1^	0.35
Timber production	Growing stock	m^3^ ha^−1^	0.25
	Harvested timber volume	m^3^ ha^−1^ 5 years	0.5
	Volume increment	m^3^ ha^−1^ 5 years	0.25
Carbon storage	Carbon in living wood	tC ha^−1^	–
	Carbon in deadwood	tC ha^−1^	–
	Carbon in harvested wood products	tC ha^−1^	–

Indicator weights were defined during meetings of stakeholders from forest policy, forest practice and forest science in Switzerland (see Blattert et al. 2020)

The carbon storage indicators comprise carbon content in living above- and belowground tree biomass as a fraction of biomass for conifers (51%) and broadleaves (48%); cf. Lamlom and Savidge (2003), in deadwood (from natural mortality and harvest residue) and in four harvested wood products (HWP). Following Blattert and Lemm (2018) and Thrippleton et al. (2021a), the harvested volume was separated for broadleaved and coniferous species in the four HWP pools (i) sawn timber, (ii) wood-based panels, (iii) paper and paperboard, and (iv) energy wood, each with individual decay rates. Estimates of tree volume and biomass used to calculate indices for timber production and above- and belowground carbon storage were calculated from the tree diameters using species-, region- and elevation-specific allometric functions from the Swiss NFI (Didion et al. 2019; Herold et al. 2019).

The biodiversity indicators include indices of tree species diversity (Shannon index, Shannon and Weaver 1949) and structural diversity (Post-hoc index, Staudhammer and LeMay 2001), the amount of deadwood volume and the number of habitat trees (DBH > 70 cm) in the stand, which are essential characteristics for structural diversity conservation (Bütler et al. 2020; Sandström et al. 2019) and are commonly used in model-based decision support systems (e.g. Blattert et al. 2017). The Post-hoc index was calculated based on DBH classes of 5 cm width and tree height classes of 2 m width.

The deadwood volume at the start of the simulation period (2016) was averaged from the observed lying and standing deadwood at NFI4 sample plots, which were classified as beech- and spruce-dominated in the development stages pole and mature, respectively. The decay of deadwood was calculated by exponential temperature-dependent decay functions specific for deciduous and coniferous trees (Mackensen et al. 2003) with a five times faster decay rate of fine deadwood (< 7 cm). More details on the indicator calculations can be found in Thrippleton et al. (2021a).

To calculate aggregated indicators of ESB provision, the indicators were scaled^2^ and averaged while considering indicators-specific and stakeholder-defined weights (Table 2) as per Blattert et al. (2020). Spearman rank correlation coefficients were calculated to analyse trade-offs and synergies among the ESB indicators. Since the simulation design is fully crossed, ANOVA models were used to quantify the effects of adaptive management and climate scenarios on the provision of ecosystem services.

## Results

### Stand development

**Fig. 3:**
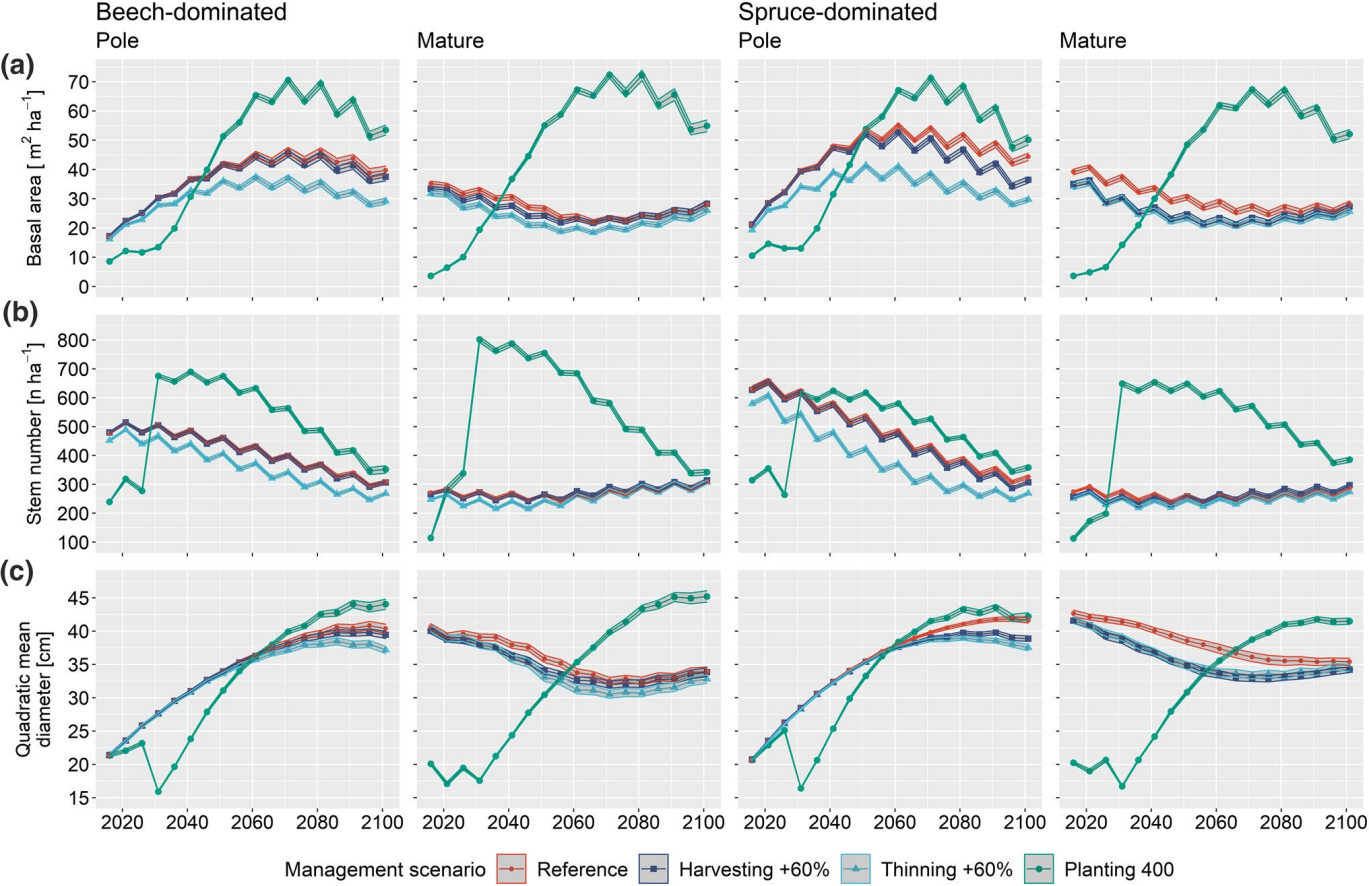
Basal area (**a**), stems per hectare (**b**) and quadratic mean diameter (**c**) under reference management, increased thinning intensity (+ 60%), increased harvesting intensity (+ 60%) and planting of 400 trees that appear with a DBH of around 12 cm in 2031 for Swiss beech- and spruce-dominated stand initialised in the pole and mature stage. The 95% pointwise confidence bands were calculated from the 20 replicates per scenario and the three climate scenarios. Note: The difference in stand characteristics between the reference and the planting 400 scenario in the first simulation decade is due to preparatory thinning

The highest peaks of basal area (up to 70 m^2^ ha^−1^), stem number (approx. 800 stems) and quadratic mean diameter (~ 44 cm) were reached in scenarios with tree planting (Fig. 3, other stand characteristics that are used as ESB indicators are presented in Fig. S4). In the planting scenarios, especially fast-growing Douglas-fir led to a considerable increase of the basal area and quadratic mean diameter, while high stem numbers (after planting) dropped with a delay due to initially low mortality and removal probabilities of the small planted trees. In contrast, increased thinning intensity clearly decreased these stand characteristics, and increased harvesting intensity slightly decreased them. In scenarios with planting, stand characteristics developed similarly in the four stand types, whereas in the other scenarios basal area and quadratic mean diameter generally decreased in simulations starting from the mature stage and increased in simulations starting from the pole stage. The uncertainty derived from the simulation replicates and simulations under the different climate scenarios was generally low, but became higher towards the end of the century. Since the confidence bands were small, the effects of the climate scenarios and simulation replications can be considered as marginal.

**Fig. 4:**
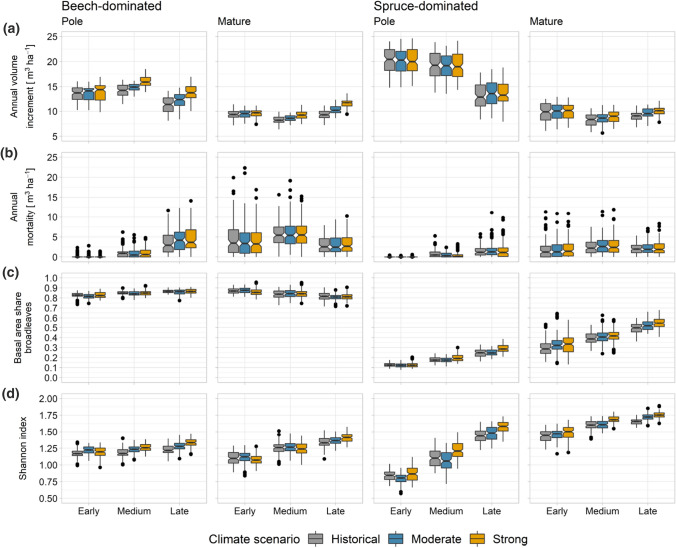
Annual volume increment (**a**), annual mortality (**b**), basal area share of broadleaves (**c**) and species diversity (Shannon index) (**d**) in beech- and spruce-dominated stands in the pole and mature development stage for three periods (early: 2016–2041; medium: 2046–2071; and late: 2076–2101) under three climate scenarios (historical, moderate (RCP4.5) and strong (RCP8.5)) and reference management

Compared to the situation under the historical climate, productivity (volume increment) increased on average by 12.5% in beech-dominated and by 3.7% in spruce-dominated stands under the strong climate warming scenario (Fig. 4a). These differences in volume increment between climate scenarios became larger towards the end of the century. In spruce-dominated stands, however, the climate scenario did not affect volume increment in the pole stage, while in the mature stage increment increased slightly in the medium and late time period (Fig. 4a). Under the moderate climate warming scenario, growth gains were generally less pronounced due to a smaller increase of temperature. Annual mortality rates were similar under all climate scenarios (Fig. 4b). The proportion of broadleaves increased in spruce-dominated stands over time, especially under the strong climate warming scenario (Fig. 4c), while in beech-dominated stands the proportion of broadleaves changed only slightly irrespective of the climate scenario. Species diversity (Shannon index) generally increased under the strong climate warming scenario in the medium and late period, while this increase was higher in simulations for the pole stage than for the mature stage (Fig. 4d). Under the moderate climate warming scenario, changes in species diversity were less pronounced.

### Development of ESB provision

**Fig. 5:**
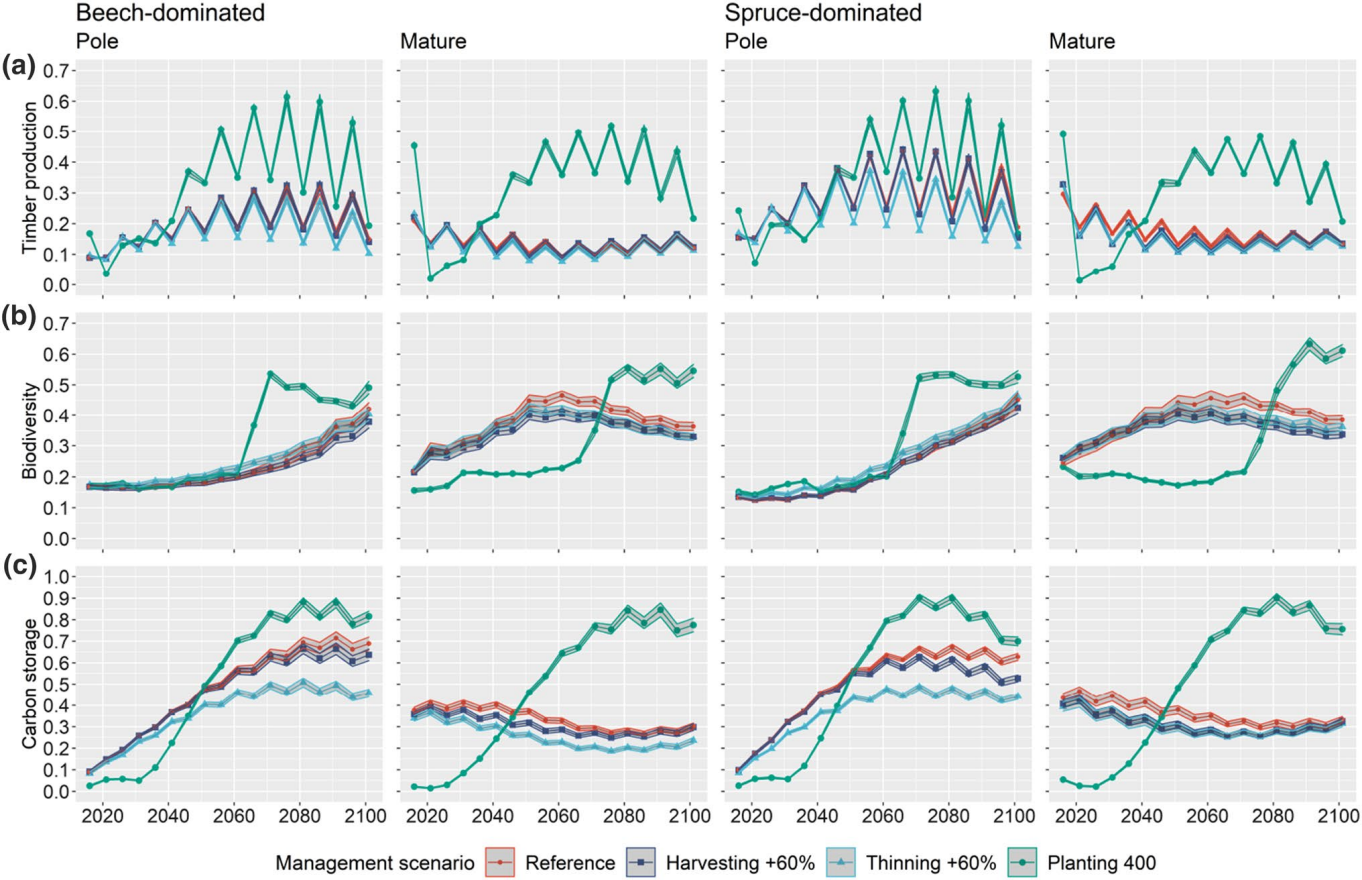
Development of timber production (**a**), biodiversity (**b**) and carbon storage (**c**) under reference management, increased thinning intensity (+ 60%), increased harvesting intensity (+ 60%) and planting of 400 trees that appear with a DBH of around 12 cm in 2031 for Swiss beech- and spruce-dominated stand initialised in the pole and mature stage. The 95% pointwise confidence bands are derived from the 20 replicates per scenario and the three climate scenarios

In the simulations for the pole stage stands, all ESB peaked in the second half of the twenty-first century when the maximum of the growing stock was reached (cf. Figure S4), while in mature stands timber supply and carbon storage typically decreased (Fig. 5). However, irrespective of the development stage, a considerable increase in all ESB was observed in the planting scenarios in the last third of the twenty-first century (green lines in Fig. 5). With planting, biodiversity peaked in the year 2076 (pole stage) and 2081–2091 (mature), i.e. when the planted trees (mainly Douglas-fir) reached the DBH to be considered as habitat trees. The combination of adaptive silvicultural interventions (e.g. tree planting and increased harvesting intensity) had no significant benefit for ESB provision compared to the individual silvicultural interventions. Harvesting in 10-year intervals as defined in the simulation setup resulted in a more or less pronounced zigzag pattern.

In scenarios with planting, timber production increased on average by 44.5% relative to the reference management, and this increase was higher in the mature stands (beech: + 98%, spruce: + 47.9%) than in the pole stands (beech: + 31.9%, spruce: + 0.2%) (Table 3; cf. Table S1). In the pole stage of the spruce-dominated stands, timber production only surpassed reference values when more than 300 trees were planted. Furthermore, planting caused biodiversity to drop in the mature stages, and at least 400 trees were necessary to increase carbon storage above reference values in the pole stages.

**Table 3:** Mean change (%) relative to the reference scenario of ESB (entire simulation period) under the three silvicultural interventions and climate scenarios in beech- and spruce-dominated stands

ESB	Management intervention	Beech-dominated		Spruce-dominated	
		Pole	Mature	Pole	Mature
Timber production	Planting	31.9 ± 4.73 (↑)	98.0 ± 6.35 (↑)	0.20± 3.67 (↑)	47.9 ± 4.65 (↑)
	Thinning	^−^8.02 ± 1.28 (↓)	^−^4.70 ± 0.70 (↓)	^−^11.2 ± 1.88 (↓)	^−^8.06 ± 0.98 (↓)
	Harvesting	^−^0.31 ± 1.03 (↓)	0.36 ± 0.66 (↕)	^−^1.36 ± 0.68 (↓)	^−^5.68 ± 0.77 (↓)
Biodiversity	Planting	18.8 ± 3.09 (↑)	^−^22.4 ± 4.25 (↑)	22.7 ± 3.18 (↑)	^−^22.8 ± 4.02 (↑)
	Thinning	4.05 ± 1.54 (↑)	^−^3.80 ± 1.77 (↓)	6.07** ± **1.33 (↑)	^−^3.38 ± 2.69 (↓)
	Harvesting	^−^2.35 ± 1.63 (↓)	^−^5.82 ± 1.98 (↓)	^−^0.51 ± 1.10 (↓)	^−^5.75 ± 2.45 (↓)
Carbon storage	Planting	^−^8.36 ± 5.76 (↑)	26.0 ± 6.13 (↑)	^−^12.7 ± 7.11 (↑)	18.0 ± 7.06 (↑)
	Thinning	^−^15.2 ± 4.54 (↓)	^−^15.9 ± 3.17 (↓)	^−^16.5 ± 4.84 (↓)	^−^9.81 ± 3.13 (↓)
	Harvesting	^−^2.09 ± 2.63 (↕)	^−^5.63 ± 1.75 (↓)	^−^4.04 ± 2.08 (↓)	^−^9.41 ± 2.68 (↓)

Standard deviations are derived from the 20 replicates per scenario and the three climate scenarios. Directions of the change over the (increasing) individual levels in brackets: Positive (↑), negative (↓) and undirected (↕)

Increasing harvesting and thinning intensity had an overall negative effect on ESB supply, with thinning generally having a greater effect. For example, a 60% increase of thinning intensity decreased timber production by 12.3% and carbon storage by 22.2%, while a 60% increase of harvesting intensity decreased timber production by 2.6% and carbon storage by 8.2%. However, thinning intensity increased biodiversity in the pole stages (on average by 5.1%), and harvesting intensity slightly increased timber production in the mature beech-dominated stand (+ 0.4%). Overall, the effects of adaptive silvicultural interventions were stronger when applied in the mature stage than in the pole stage (Table 3).

**Fig. 6:**
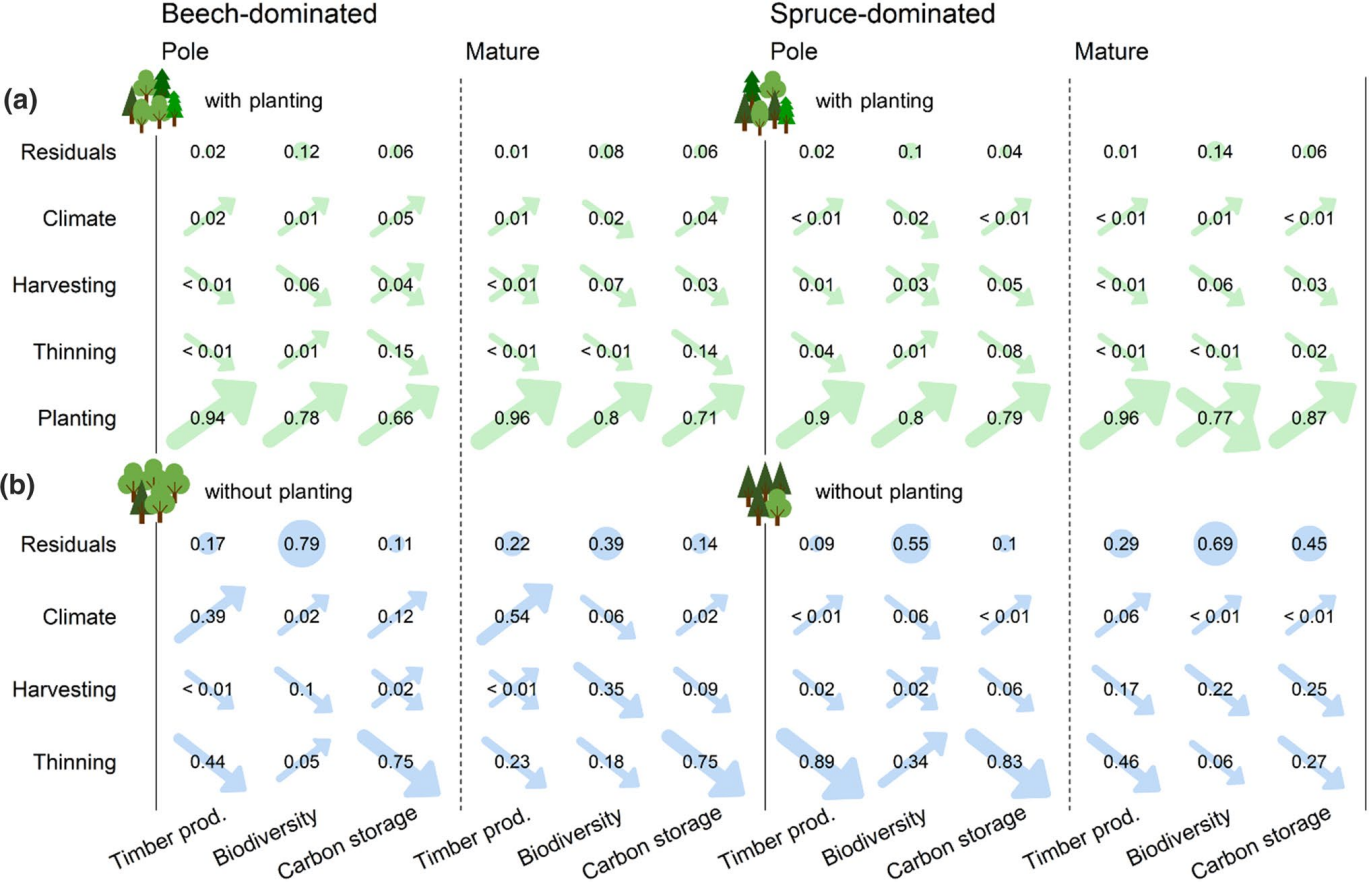
Relative sum of squares (derived from ANOVA models) explained by the error variance in the models (residuals), the adaptive silvicultural interventions and climate warming for timber production, biodiversity and carbon storage under scenarios with planting in green (**a**) and without planting in blue (**b**) in beech- (left) and spruce-dominated stands (right) initialised in the development stages pole and mature. Effect directions are indicated by arrows: Positive (↗), negative (↘) and undirected (⤨). Interactions between interventions are not presented because their contribution to explain the variance in the data was negligible

The overall large positive effects of planting on ESB provision was confirmed by ANOVA models. In models including the scenarios with planting, it explained on average 82.8% of the variance in the data, while the effect of the other interventions, climate and the residuals (error) was generally low (Fig. 6a). When the planting scenarios were excluded (Fig. 6b), the variance explained by the residuals increased substantially (especially for biodiversity), and the (mostly positive) effect of climate on timber production increased in the beech-dominated stands. The effect of increased harvesting and thinning was always negative or undirected, except for the positive effect of increased thinning on biodiversity in spruce-dominated (34%) and beech-dominated stands (5%) initialised in the pole stage. The variance explained by the interactions between interventions was negligible.

### Trade-offs and synergies among ESB

**Fig. 7:**
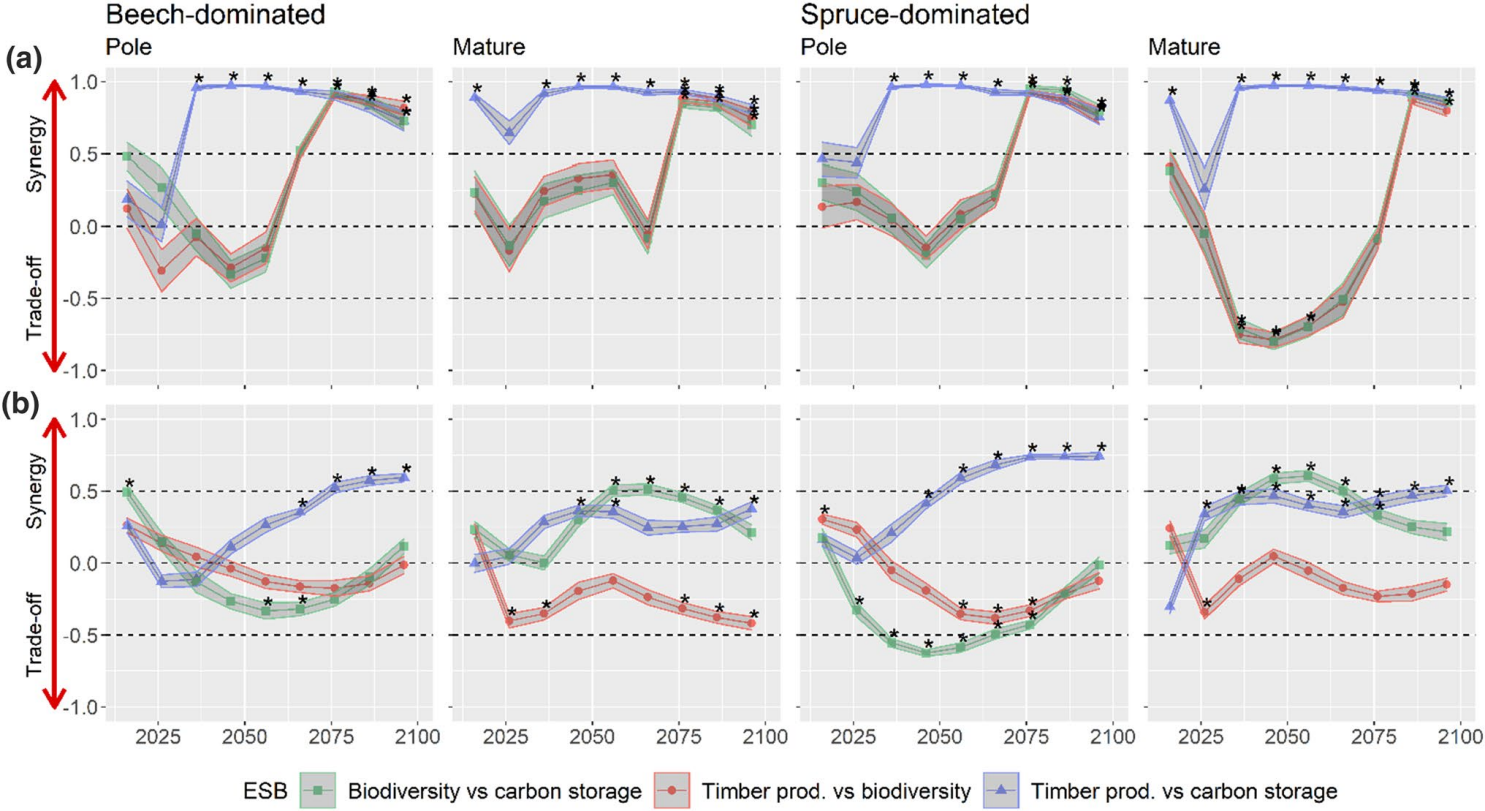
Spearman correlations as measure of trade-offs and synergies in scenarios with planting of trees (**a**) and increased thinning intensity and harvesting intensity (**b**) in beech- (left) and spruce-dominated stands (right) initialised in the development stages pole and mature. Asterisks indicate significant correlations (p < 0.05). The 95% pointwise confidence bands are derived from the 20 replicates per scenario

The planting of trees led to a pronounced synergy of timber production and carbon storage (Spearman correlation from 2031 on > 0.95) and to synergies between all ESBs towards the end of the twenty-first century, i.e. from 2076 or 2086 on (Fig. 7a). However, in the spruce-dominated stand initialised in the mature stage, planting also caused strong trade-offs between timber production and biodiversity, and carbon storage and biodiversity, in the middle of the century (Spearman correlation < −0.7).

In scenarios with increased thinning and harvesting intensity (Fig. 7b), synergies were less pronounced and persistent trade-offs between carbon storage and biodiversity and between timber production and biodiversity occurred. While in the mature stages biodiversity and carbon storage were positively related (synergy), they were generally negatively related (trade-off) in the pole stages.

## Discussion

In this study, we used a simulation-based sensitivity analysis to evaluate gradual changes of ESB provision in Swiss beech- and spruce-dominated stands under alternative intensities of adaptive silvicultural interventions and climate warming. We conducted an extensive simulation experiment to get new insights into the effect sizes and effect directions of adaptive management measures on ESB using accurate and representative stand-scale predictions.

### Effects of adaptive silvicultural interventions on ESB provision

#### Tree planting

Compared to the reference management and the two adaptive interventions of increased thinning and harvesting, tree planting (Douglas-fir, oak, and silver fir) led to the most substantial changes of ESB provision over the simulation period, irrespective of the stand type used for initialisation. The effects on ESB were always positive (with the exception of carbon storage in the pole stages and biodiversity in the mature stages) and increased with the number of trees planted. These findings correspond to results from other simulation studies conducted at different spatial scales and focused on the effect of climate change. At the plot level, Buma and Wessman (2013) showed that planting of climate-suitable species helped to maintain forest structure and carbon stocks under climate change. At the landscape scale, Hof et al. (2017) demonstrated that planting a mix of novel tree species increased resilience and carbon sequestration, and Duveneck and Scheller (2015) found that the planting of climate-suitable tree species increased aboveground biomass and species diversity under climate change. These results are supported by empirical tree-ring analyses confirming the positive effects on stand growth in drought years due to species mixtures (Vitali et al. 2018). Thus, admixing tree species that are expected to cope with climate change may unfold positive effects on ESB at different spatial scales as well as in different stand types.

Productivity and thus timber production increased considerably over the entire simulation period in the planting scenarios, whereby the effect was substantially higher in beech-dominated than in spruce-dominated stands. In the short term (20–30 years), however, ESB provision dropped and the biodiversity indicator did not increase until mid-century. These effects may be explained by (1) thinning in preparation of planting that accelerated forest restructuring in the long term, but reduced structural diversity in the short term, (2) an increased tree species diversity with the admixture of Douglas-fir, oak and silver fir, and (3) the relatively fast growth of spruce in our simulations (even under the climate change scenarios). A sufficient number of another fast-growing species, i.e. Douglas-fir, had to be planted to compensate for the losses of spruce during the initial preparation thinnings in the planting scenarios. In contrast, a mixture of just 100 trees was enough to increase timber production in slow-growing beech-dominated stands. The positive effects on productivity of admixing in monospecific stands have been reported in many studies (Messier et al. 2013; Pretzsch et al. 2010). Kelty (2006) highlighted that mixed-species forests may be even economically more attractive than monocultures due to different growth rates of the individual species and the provision of more diverse harvest products. Pretzsch and Schütze (2016) pointed out that mixed stands have higher stocking density, structural diversity, and productivity. However, arbitrary and ill-advised tree planting can have undesired or even negative consequences for ecosystems, e.g. a loss of biodiversity, and thus the planting of trees should be well planned (Brancalion and Holl 2020). We conclude that with regard to the analysed ESB indicators, tree plantings (Douglas-fir, oak and silver fir) may serve as a promising measure to ensure or even enhance ESB provision in the study area in the future, although short-term losses of ESB due to forest restructuring may occur.

Our results further suggest that especially fast-growing Douglas-fir could play a major role to secure future ESB provision. However, in Europe, the planting of Douglas-fir, as a non-native species, is controversially discussed. On the one hand, Douglas-fir is fast growing, has a high drought tolerance and good wood quality (Spiecker et al. 2019). On the other hand, there are concerns on Douglas-fir’s invasive potential, its competitive interaction with native species, and that it may carry novel pathogens. However, a recent national (Germany) inventory-based risk assessment (Bindewald et al. 2021) showed that the risk of invasiveness of Douglas-fir is low, and also Tschopp et al. (2015) found no negative impacts of Douglas-fir on biodiversity in Central Europe, although generalists rather than specialists are supported by Douglas-fir. Thus, a partial replacement of spruce with Douglas-fir at low- to mid-elevations and its cautious admixture in beech stands appears acceptable also from a biodiversity perspective.

Native oak and silver fir are considered relatively drought-tolerant and may thus cope well with climate change in Central Europe (Sáenz-Romero et al. 2017; Vitasse et al. 2019). Huber et al. (2021) found that the admixture of native drought-tolerant species (including silver fir and oak) supports carbon storage and thus climate change mitigation, and also Baumbach et al. (2019) recommend the admixture of silver fir in vulnerable beech forests in Central Europe. In sum, we suggest that the benefits of planting a mixture of Douglas-fir, oak and silver fir may be higher than the risks. However, tree planting is usually associated with high costs for planting material and labour (Spiecker et al. 2019), even if the costs can be reduced through appropriate planting methods, e.g. cluster planting (Saha et al. 2017). In our simulations the planted trees appeared after 15 years of simulation with a DBH of around 12 cm (100, 200, 300 or 400 trees) and potential losses due to competition or disturbances during the establishment phase of the saplings were only roughly estimated. These costs and risks must be carefully balanced with the benefits of admixing stands with Douglas-fir, oak and silver fir.

#### Increased thinning and harvesting intensity

Increased thinning and harvesting intensity were aimed to facilitate forest restructuring and support species mixture in our simulations. Within a relatively short time frame such strategies promise to increase stand resilience against disturbances (Bolte et al. 2009; Zimová et al. 2020). However, our results show that thinning and harvesting intensity typically reduced ESB provision (except for timber production in mature beech-dominated stands and biodiversity in pole stands, as discussed below). These results correspond to findings by Zimová et al. (2020) who suggest that the shortening of the rotation length (in our study represented by an increased harvesting intensity) may have negative consequences on ESB provision including reduced carbon storage. Felton et al. (2017) simulated a loss of habitat features due to shortened rotation lengths in northern production forests. Further, Noss (2001) pointed out that a shortened rotation period bears the risk of soil degradation and may facilitate species invasions. In contrast, extended rotation forestry may have direct positive effects on ESB provision by restoring old-growth structures and facilitating high productivity (D’Amato et al. 2010; Silver et al. 2013). Steenberg et al. (2011) found that excluding younger trees from harvesting was most efficient for maintaining timber supply, which corresponds to our finding that timber production decreased under higher thinning intensities of beech and spruce across all diameter classes including small trees. Thus, when considering increased thinning and harvesting intensity as an adaptation strategy, the risks and potential drawbacks in terms of ESB provision must be taken into account.

The loss of ESB provision under intensified thinning and harvesting reflects the recent growing stock reduction on the Swiss Plateau captured by the NFI data used to parameterise the harvesting model (survey GLM) that was then applied in the simulations. Growing stocks decreased from 441 m^3^ ha^−1^ in 1995 to 380 m^3^ ha^−1^ in 2013 in the Swiss Plateau region (Camin et al. 2015). In our simulations, an increase in thinning and harvesting intensity led to a further decrease in the growing stocks in the study area and caused the loss of ESB supply in comparison to the reference scenario. Overall, our results suggest that in the investigated study region (covering the Swiss Plateau) an increase in thinning and harvesting intensity cannot be the universal solution, but may only be suitable locally where current growing stocks and the risk for windthrow and bark beetle disturbance events are particularly high.

### Model response to climate scenarios

We found a small positive effect of climate warming on ESB provision. This effect was higher in beech-dominated than in spruce-dominated stands. Reflecting the empirical basis of SwissStandSim, rising annual mean temperatures slightly elevated the productivity of beech and thus facilitated forest restructuring and increased biodiversity especially in spruce-dominated stands. These findings correspond to retrospective studies from Forrester et al. (2021) who reported an increased tree growth in beech- and spruce-dominated stands in Switzerland that was mainly temperature-driven, and Pretzsch et al. (2014) who demonstrated that growth of beech and spruce accelerated in Central Europe over the last 100 + years. In our simulations, this productivity boost in turn led to an increase in timber production under the climate scenarios, which was also reported in other forward-looking studies (e.g. Joyce et al. 1995; Steenberg et al. 2011).

In particular, spruce outside its natural distribution range (Honkaniemi et al. 2020; Lévesque et al. 2013), but also beech (Jump et al. 2006; Rohner et al. 2021) has been repeatedly reported to be highly susceptible to drought spells and disturbances such as windthrow and bark beetle infestation, which are all likely to occur more frequently and become more severe in the future (Seidl et al. 2016). However, such drought- and disturbance-induced growth declines and mortality were not accounted for in our simulations (see Sect. Methodological considerations below). Considering them would likely affect our simulation results in the sense that forest restructuring under the planting and intensified thinning and harvesting scenarios would be even faster. This in turn would enhance the long-term synergetic effects for ESB provision (cf. Section Trade-offs and synergies among ESB), but would also reinforce short-term trade-offs. For example, increased tree mortality may benefit biodiversity through higher deadwood availability and higher stand structural complexity (Cours et al. 2021; Winter et al. 2015), while carbon storage and timber production may be negatively affected (Thom and Seidl 2016). While our results of tree responses to climate warming correspond to previous observations, their interpretation needs to keep in mind the potential effects of climatic extremes and disturbances, which our model was unable to capture.

### Trade-offs and synergies among ESB

Tree plantings enabled pronounced synergies between all ESB by the end of the twenty-first century, while increased thinning and harvesting intensity more often led to trade-offs between timber production and biodiversity, and carbon storage and biodiversity. This positive effect of planting can be explained by a number of reasons: a) After 50–65 years of simulation, planted Douglas-firs reached the diameters to be classified as habitat trees and immediately increased the biodiversity value. b) Douglas-fir was generally the fastest-growing species in our simulations and thus increased both timber production (harvestable diameters were reached earlier) and carbon storage (living biomass accumulated faster) in comparison with stands without Douglas-fir. c) Increasing the number of planted trees further increased the synergetic effects described in a) and b), while interspecific competition remained low due to thinnings in preparation of planting. d) Planted Douglas-fir, silver fir and oak trees grew at different speeds, which increased biodiversity.

These results correspond to findings from Mina et al. (2017) who showed that trade-offs and synergies among ESB were sensitive to the management strategy. Depending on site-specific growth conditions, an increase or decrease in management intensity facilitated trade-offs, e.g. between timber production and carbon storage, and synergies, e.g. carbon storage and protection against natural hazards. Under high management intensities, a trade-off between timber supply and biodiversity was often reported (Dickie et al. 2011; Duncker et al. 2012). In contrast, several studies report synergetic effects of planting on ESB (Buma and Wessman 2013; Duveneck and Scheller 2015). The positive implications of plantings on ESB underline the benefits of well-planned admixing of climatically suited and fast-growing tree species in the study area and at other suitable sites.

A striking result was that in the planting scenarios, trade-offs (carbon storage vs biodiversity and timber production vs biodiversity) occurred in the mature spruce-dominated stand only. This may be due to the generally higher productivity of spruce compared to beech in the study area (Pretzsch et al. 2010). Under low planting intensity (100 trees), more mid-size (DBH 12–30 cm) spruce trees remained in the stand than under high planting intensity (400 trees), so that spruce contributed more to the productivity in the early to mid-phase of the simulation. However, in the long term these trade-offs, also reported in other studies (Lafond et al. 2017; Turner et al. 2013), disappeared as the planted trees overcompensated for the losses of spruce. Further, considering the high vulnerability of spruce to changing climate and disturbance regimes, especially at low elevations (Honkaniemi et al. 2020), the temporary disadvantages of tree planting may disappear earlier or even completely. This suggests that plantings in mature spruce-dominated stands can be useful despite the temporary trade-offs, although admixing in younger stands may be preferable in view of biodiversity conservation.

### Methodological considerations

Simulation-based sensitivity analysis is a valuable tool to go beyond the evaluation of single scenarios (Lafond et al. 2015). Many simulation studies use expert- or literature-based management storylines that are translated into scenarios with fixed intervention levels (e.g. planting of 100 climate-suitable trees) (Halofsky et al. 2017; Hilmers et al. 2020; Seidl et al. 2011; Temperli et al. 2012). However, considering only a specific intervention level may produce incomplete or even misleading results due to the potentially high uncertainty of intervention level selection. In contrast, a sensitivity analysis based on a large number of scenarios with alternative intervention levels allows for investing gradual changes, multiple interactions and tipping points or even mathematically optimised levels of the adaptation interventions (e.g. González et al. 2005; Lafond et al. 2017). This enables a comprehensive evaluation of management strategies by means of effect sizes and directions. However, multi-scenario simulations are computationally intensive, which complicates the application of forest development models with highly detailed process representation and/or large area coverage (Irauschek et al. 2017; Petter et al. 2020) for such analyses. In this respect, computationally efficient empirical models such as SwissStandSim (Zell et al. 2020) have an advantage. Additionally, SwissStandSim is temperature sensitive and provides a comprehensive representation of individual-tree-based stand development (growth, mortality, ingrowth, harvest) parameterised for different forest types in Switzerland and thus outperforms other empirical modelling approaches in the study region (e.g. Trasobares et al. 2016).

Simulation studies are subject to a number of uncertainties and model assumptions. Accordingly, the results of our study must be seen in the light of several limitations. The climate sensitivity of empirical models is limited by the empirical data used. Leaving the so-called “climate space” under climate change scenarios, i.e. leaving the multidimensional climate conditions of the area (Ohlemüller 2011) in which the calibration data were collected, may lead to simulated forest development that is not supported by the model assumptions. In our simulations, temperatures under the climate change scenarios exceeded the temperature range covered by the growth and mortality models in the second half of the twenty-first century. This resulted in a moderate growth boost for beech. Because the simulated growth response to rising temperature can be explained ecologically (Way and Oren 2010), we consider it plausible even though it is outside the parameterisation range.

Mortality rates in our simulations did not respond to the increased temperature averages and thus the dryer conditions under the climate scenarios due to the following reasons. 1) Drought has not been a decisive factor for tree mortality in the Growth-And-Yield plots. These plots have been set up on productive, presumably rather moist, sites suitable for the production of high-quality timber. The empirical basis of SwissStandSim would have to be extended to plot data from sites with poorer water availability, where past exposure of spruce and beech to drought has been recorded (Lévesque et al. 2013; Rohner et al. 2021). 2) The model’s lacking sensitivity to drought may partly be due to the use of 5-year averages of spring (March-June) temperature and precipitation for the calculation of the moisture index (Zell et al. 2020). While this index was suitable to explain the patterns found in the Growth-And-Yield data, it does not account for extreme summer droughts that may occur in individual years. Such drought spells may be more important in controlling drought-induced mortality in beech and spruce forests than the shift in average drought conditions (Rohner et al. 2021; Schuldt et al. 2020). Future model amendments should focus on including bioclimatic predictor variables that capture extreme drought events in mortality models (Thrippleton et al. 2021b). 3) Mortality due to disturbances could not be differentiated from other mortality reasons (forest management, self-thinning, etc.) in the growth and yield network data. Hence, climate change-enhanced disturbances were not explicitly simulated. However, salvage cutting may have influenced our estimates of harvesting and thinning rates.

Further limitations are related to the selection of indicators used for the scenario evaluation. The analysis was restricted to the three ESB timber production, carbon storage and biodiversity. Considering other ESB such as recreation (Edwards et al. 2012) and advanced weighting tools such as multi-criteria decision analysis could broaden the analysis (Blattert et al. 2020). Integrating the effects of substituting fossil fuel intensive products with timber in our assessment of carbon storage could further extend the portfolio of ESB indicators (Leskinen et al. 2018). Accounting for substitution effects may, in contrast to our study, lead to the result that increased thinning and harvesting benefit carbon storage as long as forest productivity remains high (Verkerk et al. 2020). Also, our approach did not account for the financial aspects of the silvicultural interventions. In particular, the relatively high costs for planting and tending need to be considered to determine whether planting new tree species to enhance ESB provision is feasible in specific cases. Additionally, investigating the effects of the time point of planting and repeated planting regimes (once and fixed in our study) may improve the realism of the recommendations for forest practice. The selection and weighting of indicators had a great influence on the evaluation of ESB. For example, the biodiversity indicator “habitat trees” substantially increased biodiversity towards the end of the century in the planting scenarios, although the actual effect of the planted trees on biodiversity is more complex and should also account for the role of the species in the stands. For example, oaks are considered to be particularly valuable for biodiversity (Mölder et al. 2019), whereas this is much less the case for Douglas-fir. Integrating these aspects in our indicator framework would complement the picture of ESB provision that can be drawn from the simulation results.

## Conclusions

Our results show that planting climatically suited and fast-growing tree species is most effective to maintain and enhance ESB provision in the long term by facilitating synergies among ESB, but benefits of ESB need to be balanced against planting and tending costs. Further research using productivity models for planting and tending operations (e.g. Holm et al. 2020) may support such considerations. The behaviour of the empirical model under climate change scenarios, e.g. growth gains of beech under increasing annual mean temperatures and thus mostly positive effects on ESB provision, pinpoints the need for better accounting the effects of drought and disturbances such as windthrow and bark beetles to improve the realism of such scenario simulations under climate change. Integrating disturbance regimes with stochastic approaches into empirical model frameworks may allow for such assessments (e.g. Kunstler et al. 2013) while maintaining the computational efficiency and regional accuracy of empirical models.

We demonstrated that tree plantings and preparatory thinnings are more beneficial when applied in the mature stage than in the pole stage, as interventions in this development stage may fit better into the succession of the stands. Further, we identified and discussed risks and potential drawbacks of intensified management on ESB provision in the study region. Our results are representative of typical low- to mid-elevation spruce- and beech-dominated stands in Switzerland, but actual management needs to account for local site conditions, forest composition and structure, and ESB demands. However, the discussed findings on the application of adaptive management tools can be particularly useful for federal management plans and federal policy making (e.g. BAFU 2021).

In summary, we provide a simulation-based sensitivity analysis framework with accurate stand-level predictions that go beyond the case study level and are representative at the regional scale. Using a fully crossed experimental design, the sizes and directions of silvicultural intervention effects as well as trade-offs and synergies among ESB became directly assessable. This is a step forward to improve management guidance in the face of multiple options for forest adaptation to climate change.

## Supplementary Information

Below is the link to the electronic supplementary material.

Supplementary file1 (DOCX 3618 KB)

## Footnotes

1 The two removal probabilities (21.8% and 2.4%) can be compared based on: 1—0.218 ≈ (1—0.024)^10^.

2 *x*’ = ( *x* – min(*x*)) / ( max(*x*) – min(*x*)).
