# Supplementary material for: Tree species admixture increases ecosystem service provision in simulated spruce- and beech-dominated stands
Source: Eur J For Res. 2022 Jul 2;141(5):801–20. doi: 10.1007/s10342-022-01474-4 (PMC9519722; doi:10.1007/s10342-022-01474-4)
Supplement: Supplementary file 1 — Supplementary file1 (DOCX 3618 KB) [file 10342_2022_1474_MOESM1_ESM.docx]

**Supporting Information to “Tree species admixture increases ecosystem service provision in simulated spruce- and beech-dominated stands”**

Reinhard Mey, Jürgen Zell, Esther Thürig, Golo Stadelmann, Harald Bugmann, Christian Temperli

**S1 Light- and temperature dependent ingrowth in SwissStandSim**

Test simulations showed that the basic ingrowth process implemented in SwissStandSim (Zell et al. 2020) was not able to account for sufficient ingrowth after heavy thinning (or disturbance events) and in long-term simulations (>50 years). Thus, a new light- and temperature-dependent ingrowth process (Fig. S1) was introduced for simulations presented in this manuscript.

The new ingrowth process combines a light- and temperature-dependent part: Single-tree diameter and species information are used to calculate the available light at the forest floor (green part of Fig. S1). Then, a negative-binomial model (details below) is used to predict the number of ingrowing trees as a function of the available light at the forest floor. The dominant diameter and the mean winter temperature are used to select five samples from the fourth campaign of the Swiss NFI (yellow part of Fig. S1). Summary statistics from these samples are then used to derive a diameter distribution and the species composition using the method from Mey et al. (2021). From this diameter distribution, diameters for each ingrowing stem are randomly selected until the previously determined number of ingrowing trees is reached. Thereby, the routine only selects stems larger than 12 cm in diameter and smaller than the diameter that a 12 cm stem can grow to within the five-year time step, according to the SwissStandSim-inherent growth function. The species identity of the ingrowing stem is also randomly selected based on the species composition determined from the NFI data. This process is repeated every five-year time step.

The negative-binomial model for the number of ingrowing trees was derived using samples from the four consecutive inventories of the Swiss NFI (NFI1 to NFI4). In a first step, the available light at the forest floor was calculated (based on the green part of Fig. S1) for each NFI sample of tree diameters and species. Then a negative-binomial model (R package glmmTMB) was fitted to explain the number of ingrowing trees (determined by the Swiss NFI field team at each plot) by the available light at forest floor of the previous inventory period. A negative-binomial model was fitted separately for plots classified as beech-dominated (basal area of beech >50%), spruce-dominated (basal area of spruce >80%) and other forests.


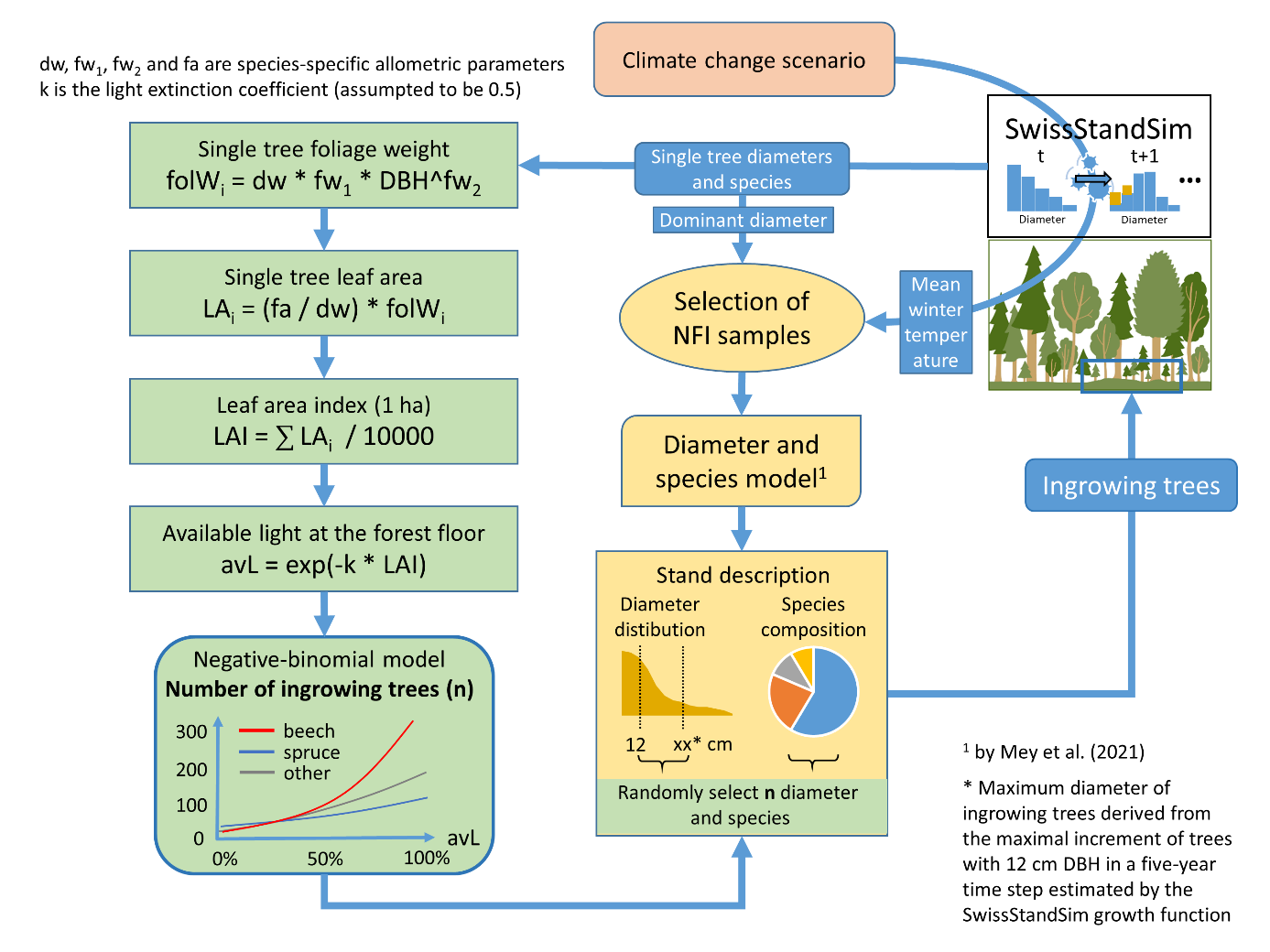


**Fig. S1** New light- and temperature-dependent ingrowth process of SwissStandSim. In green the light-dependent calculation of the number of ingrowing trees based on the available light (avL) at the forest floor (Schumacher 2004). In yellow the temperature-dependent estimation of stand descriptions using the approach by Mey et al. (2021)

Model formulation of the negative-binomial model (in R):

*glmmTMB(formula = number of ingrowing trees ~ available light previous inventory + offset(log(time between two consecutive inventories * plot area/10000)) + (1 |inventory number), data = sample data, family = nbinom2, se = TRUE, ziformula = ~0, dispformula = ~1)*

**S2 BAU model**

Model formulation of the business as usual harvesting model (in R):

*survey design <- svydesign(id = ~plot number, strata = ~inventory number, weights = ~representation factor of individual trees, nest = TRUE, data = data)*

*svyglm(formula = harvest (0, 1) ~ diameter at breast height * tree species group + I(diameter at breast height^2) + number of stems per hectare * basal area + I(number of stems per hectare^2) + increment + I(increment^2) + slope + ecoregion + elevation + accessibility + storm event + forest ownership + time between two consecutive inventories, design = survey design, family = quasibinomial())*

**S3 Stand development under business as usual management and adaptive silvicultural interventions**


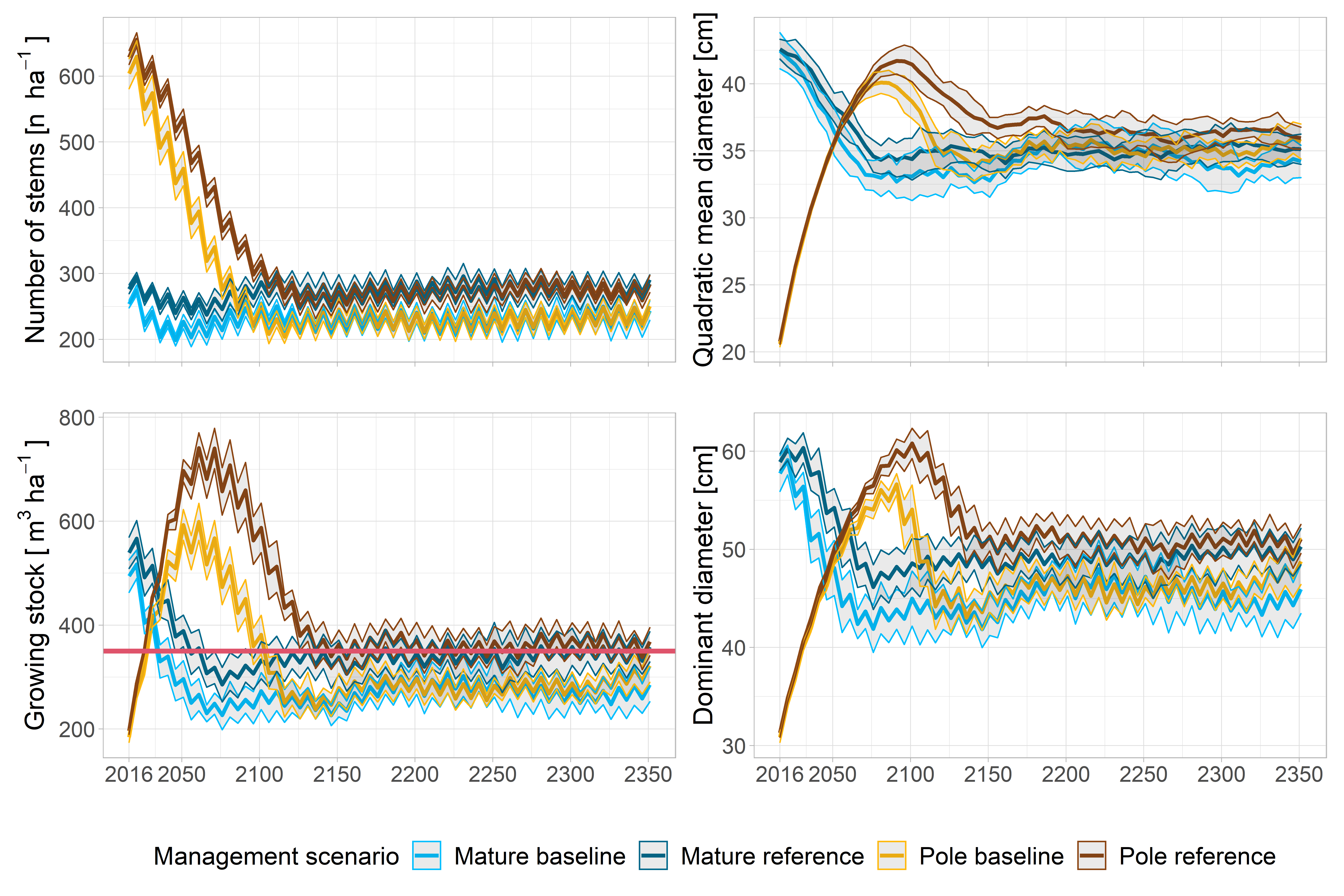


**Fig. S2** Stand attributes of spruce-dominated stands initialised in pole and mature stages under baseline management, reference management (baseline removal probability reduced by 25%) and historical climate over 300 years. The 95% pointwise confidence bands are derived from the 20 simulation replicates. The red horizontal line indicates the current growing stock (2009/13) at the Swiss plateau (Camin et al. 2015)


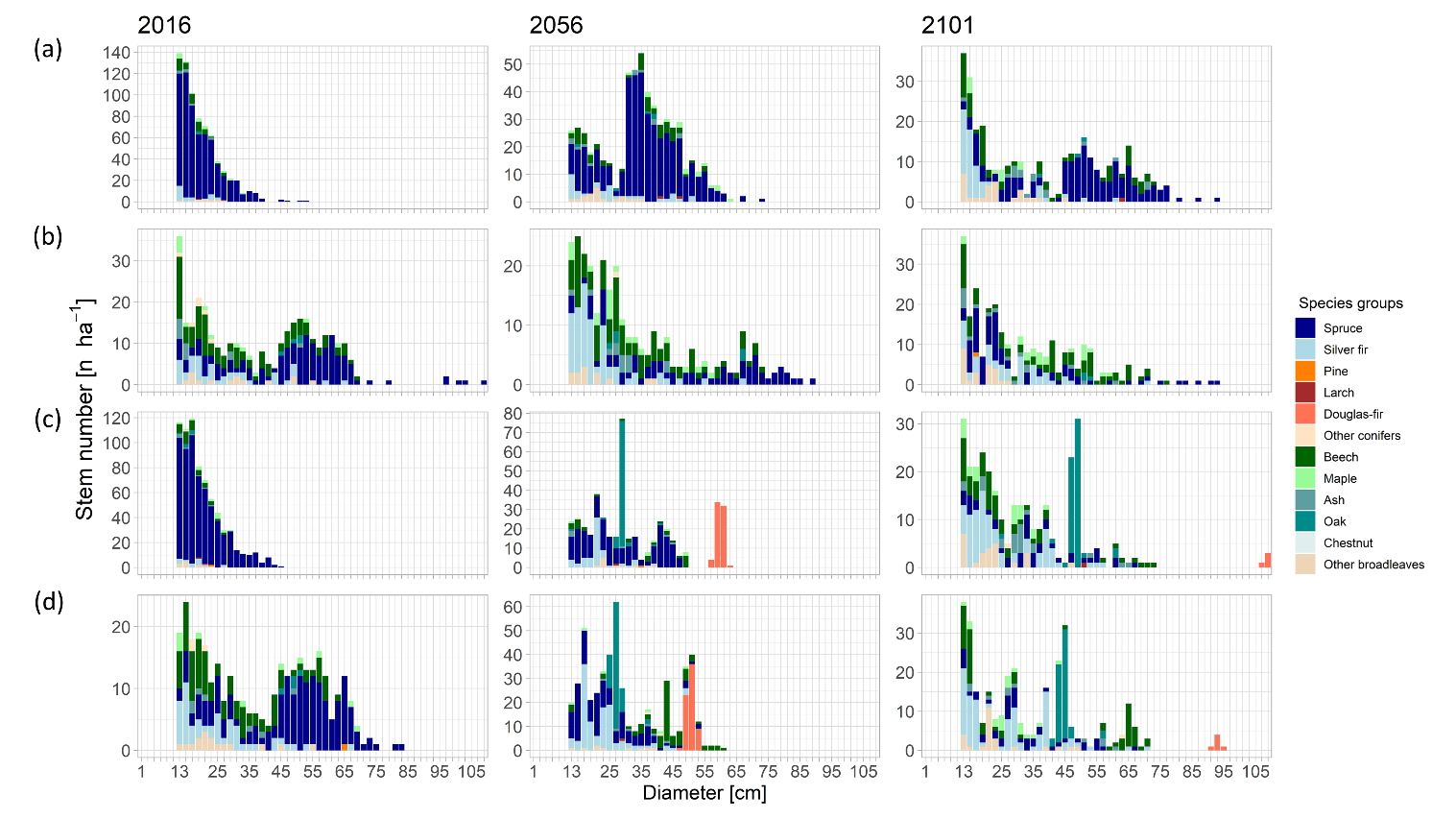


**Fig. S3** Example realisation of the temporal development (2016, 2056 and 2101) of the diameter distribution and species composition of spruce-dominated stands in the development stages pole and mature under reference management (a and b) and adaptive management with planting (ingrowth of 200 trees in 2031) and 30% increased thinning and harvesting intensity (c and d). All stands convert towards inverse J-shaped diameter distributions of continuous cover stands





**Fig. S4** Growing stock, harvested volume, volume increment, Shannon index, Post-hoc index, deadwood volume, habitat trees (DBH > 70 cm), carbon in living wood, carbon in deadwood and carbon in harvested wood products under reference management, increased thinning intensity (+60%), increased harvesting intensity (+60%) and planting of 400 trees that appear with a DBH of around 12 cm in 2031 for Swiss beech- and spruce-dominated stand initialised in the pole and mature stage. The 95% pointwise confidence bands were calculated from the 20 replicates per scenario and the three climate scenarios. Note: The difference in stand characteristics between the reference and the planting 400 scenario in the first simulation decade is due to preparatory thinning

**S4 Effects of adaptive silvicultural interventions on ESB provision**

**Table S1** Mean change (%) relative to the reference scenario of ESB (entire simulation period) under the four levels (planting: 100, 200, 300 and 400 trees; thinning and harvesting: removal probability increased by 15%, 30%, 45% and 60%) of the three silvicultural interventions and climate scenarios in beech- and spruce-dominated stands

| ESB |  | Beech-dominated | | | | | | | |
| --- | --- | --- | --- | --- | --- | --- | --- | --- | --- |
|  |  | Pole | | | | Mature | | | |
|  |  | Level 1 | Level 2 | Level 3 | Level 4 | Level 1 | Level 2 | Level 3 | Level 4 |
| Timber production | Planting | 2.8 | 23.2 | 42.0 | 59.5 | 63.0 | 89.0 | 109.5 | 130.6 |
|  | Thinning | -3.8 | -6.5 | -9.4 | -12.4 | -2.3 | -4.1 | -5.1 | -7.3 |
|  | Harvesting | -0.1 | -0.3 | -0.4 | -0.4 | 0.2 | 0.6 | 0.3 | 0.3 |
| Biodiversity | Planting | 2.6 | 15.7 | 25.0 | 31.9 | -32.3 | -24.1 | -18.8 | -14.3 |
|  | Thinning | 2.5 | 3.0 | 5.0 | 5.8 | -1.0 | -3.0 | -5.1 | -6.1 |
|  | Harvesting | -0.4 | -1.9 | -2.6 | -4.4 | -2.1 | -5.2 | -6.9 | -9.0 |
| Carbon storage | Planting | -24.8 | -12.6 | -3.1 | 7.0 | 5.8 | 20.6 | 33.4 | 44.4 |
|  | Thinning | -6.9 | -12.2 | -18.5 | -23.4 | -7.2 | -13.7 | -18.6 | -24.2 |
|  | Harvesting | -1.0 | -1.9 | -1.8 | -3.6 | -2.1 | -5.0 | -6.6 | -8.8 |
| ESB |  | Spruce-dominated | | | | | | | |
|  |  | Pole | | | | Mature | | | |
|  |  | Level 1 | Level 2 | Level 3 | Level 4 | Level 1 | Level 2 | Level 3 | Level 4 |
| Timber production | Planting | -19.6 | -5.1 | 7.7 | 17.9 | 19.4 | 41.4 | 58.9 | 71.8 |
|  | Thinning | -4.8 | -9.1 | -13.1 | -17.7 | -4.2 | -7.1 | -9.0 | -11.9 |
|  | Harvesting | -0.4 | -0.8 | -1.8 | -2.4 | -3.5 | -4.8 | -6.8 | -7.7 |
| Biodiversity | Planting | 7.8 | 19.4 | 28.1 | 35.3 | -27.9 | -23.0 | -20.1 | -20.3 |
|  | Thinning | 2.7 | 4.8 | 7.2 | 9.6 | -0.6 | -3.1 | -3.8 | -6.1 |
|  | Harvesting | -0.2 | -0.1 | -1.0 | -0.7 | -2.8 | -4.9 | -6.5 | -8.8 |
| Carbon storage | Planting | -33.0 | -18.0 | -4.9 | 5.3 | -9.5 | 11.7 | 29.0 | 40.8 |
|  | Thinning | -6.8 | -13.8 | -19.6 | -25.7 | -4.0 | -8.9 | -10.8 | -15.5 |
|  | Harvesting | -1.4 | -2.6 | -5.2 | -7.0 | -5.9 | -8.1 | -10.3 | -13.3 |

**S5 Climate scenarios**


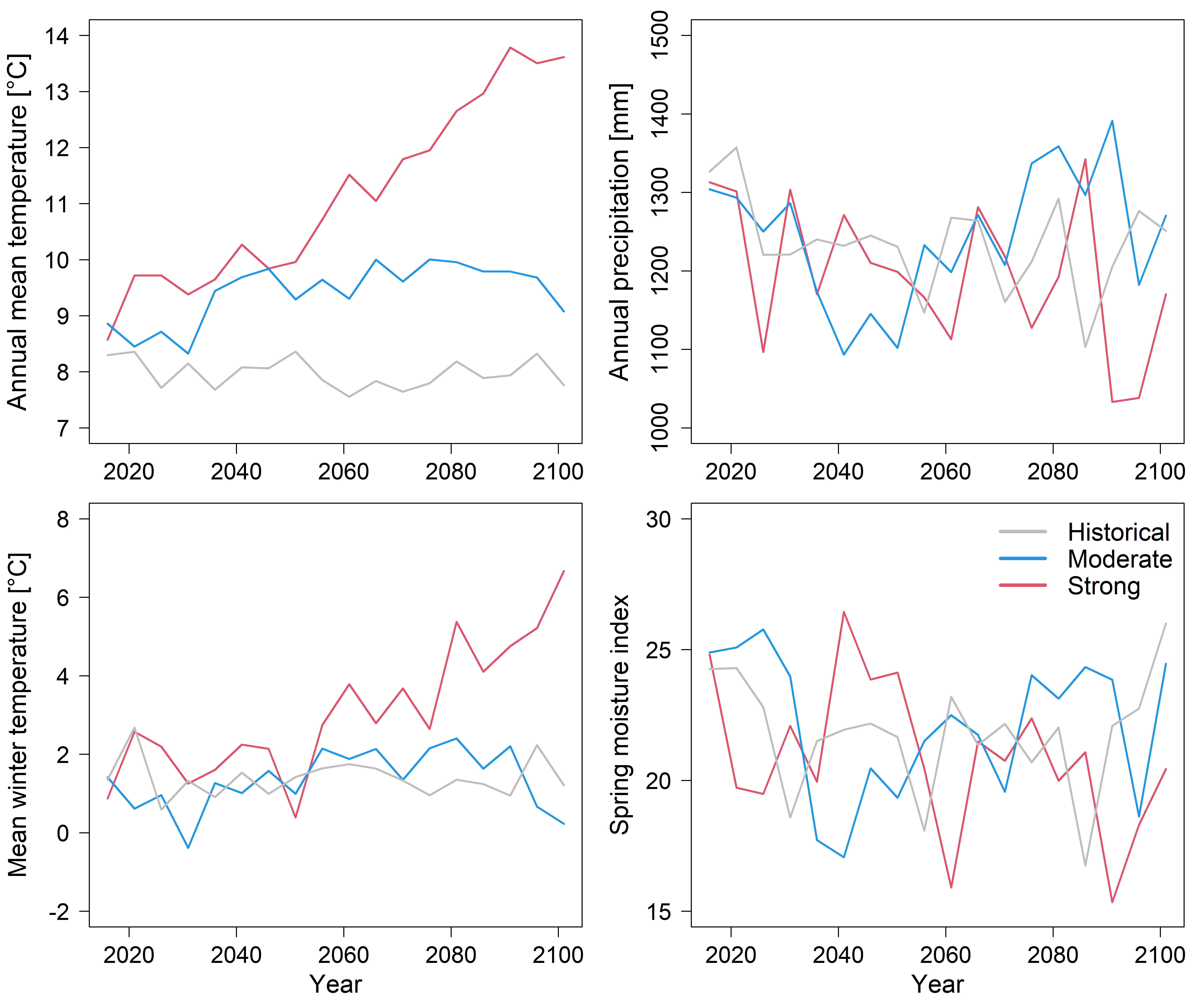


**Fig. S5** Annual mean temperature, annual precipitation, spring moisture index (Mar-Jun) and mean winter (Dec-Feb) temperature averaged over the NFI4 plots used to derive the initial stands (cf. Fig. 1) under a historical (1986-2015), a moderate (RCP4.5) and a strong (RCP8.5) climate change scenario

**References**

Camin P, Cioldi F, Röösli B (2015) Holzvorrat. In: Rigling A, Schaffer HP (eds) Waldbericht 2015. Zustand und Nutzung des Schweizer Waldes. Bundesamt für Umwelt, Eidg. Forschungsanstalt für Wald, Schnee und Landschaft WSL, Bern, Birmensdorf, pp 32–33

Mey R, Stadelmann G, Thürig E, Bugmann H, Zell J (2021) From small forest samples to generalised uni‐ and bimodal stand descriptions. Methods Ecol Evol 12:634–645. https://doi.org/10.1111/2041-210X.13566

Schumacher S (2004) The role of large-scale disturbances and climate for the dynamics of forested landscapes in the European Alps. Dissertation, ETH Zurich. https://doi.org/10.3929/ethz-a-004818825

Zell J, Nitzsche J, Stadelmann G, Thürig E (2020) SwissStandSim: ein klimasensitives, einzelbaumbasiertes Waldwachstumsmodell. Schweizerische Zeitschrift für Forstwesen 171:116–123. https://doi.org/10.3188/szf.2020.0116
